# COVID-19-Associated Hyper-Fibrinolysis: Mechanism and Implementations

**DOI:** 10.3389/fphys.2020.596057

**Published:** 2020-12-16

**Authors:** Giris Jacob, Anat Aharon, Benjamin Brenner

**Affiliations:** ^1^Medicine F and Recanati Research Center, Tel Aviv Medical Center, Sackler Faculty of Medicine, Tel Aviv University, Tel Aviv, Israel; ^2^Hematologic Research Laboratory, Hematologic Department, Tel Aviv Medical Center, Sackler Faculty of Medicine, Tel Aviv University, Tel Aviv, Israel; ^3^Coagulation Research Laboratory Unit, Department of Hematology, Rambam Medical Center, Rappaport Faculty of Medicine, Technion – Israel Institute of Technology, Haifa, Israel

**Keywords:** SARS-CoV-2, COVID-19, fibrinolysis, coagulation, tranexamic acid

## Abstract

The emerging novel coronavirus disease (COVID-19), which is caused by the SARS-CoV-2 presents with high infectivity, morbidity and mortality. It presenting a need for immediate understanding of its pathogenicity. Inflammation and coagulation systems are over-activated in COVID-19. SARS-CoV-2 damages endothelial cell and pneumocyte, resulting in hemostatic disorder and ARDS. An influential biomarkers of poor outcome in COVID-19 are high circulating cytokines and D-dimer level. This latter is due to hyper-fibrinolysis and hyper-coagulation. Plasmin is a key player in fibrinolysis and is involved in the cleavage of many viruses envelop proteins, including SARS-CoV. This function is similar to that of TMPRSS2, which underpins the entry of viruses into the host cell. In addition, plasmin is involved in the pathophysiology of ARDS in SARS and promotes secretion of cytokine, such as IL-6 and TNF, from activated macrophages. Here, we suggest an out-of-the-box treatment for alleviating fibrinolysis and the ARDS of COVID-19 patients. This proposed treatment is concomitant administration of an anti-fibrinolytic drug and the anticoagulant.

## Introduction

The emerging novel coronavirus disease (COVID-19), which is caused by the SARS-CoV-2, presents with high infectivity, morbidity, and mortality. The pressing need for understanding the virus’ pathogenicity and its interaction with the body’s biologic defense systems are required (Li et al., 2020). The inflammatory response and the coagulation system frequently join forces to build an effective defense against an assaulting pathogen (Arneth, 2019). Interactions between these two systems offer potential opportunities for novel therapeutic modalities. Unusually high circulating D-dimer (DDI) levels are a main predictor of poor outcome, and indicate that the coagulation and fibrinolytic systems are overactive in COVID-19 (Tang et al., 2020b; Zhou et al., 2020). This review highlights the relationship between virus infectivity and the fibrinolytic system, and suggests a potential new therapeutic modality to mitigate the virus’ infectivity in COVID-19.

### Etiologic-Pathogenicity of COVID-19

SARS-CoV-2 is a RNA beta-coronavirus of zoonotic origin, and is closely related to the SARS-CoV and MERS-CoV, according to whole genome sequencing (Shirato et al., 2020; Zhou et al., 2020). The virus is highly infective and respiratory droplets are the main route of its transmission between humans (Ong et al., 2020).

The basic pathogenesis of COVID-19 shares common characteristics with that of SARS and MERS. From a clinical perspective, the airways and lungs are the most affected organs (Pan et al., 2020). Autopsies of COVID-19 patients reveal that the vascular bed is also severely affected (Fox et al., 2020). This specific tropism of SARS-CoV-2 for epithelial cells of the lungs and vascular systems could explain its infectivity.

The spike protein on the surface of the glycoprotein envelope of SARS-CoV-2 comprises two domains: a receptor-binding domain (S1), which binds with high affinity to the angiotensin-converting enzyme 2 (ACE2) receptor on the membranes of human pneumocytes and vascular endothelial cells, (Hamming et al., 2004; Whittaker and Millet, 2020; Zhang et al., 2020) and an S2 domain for anchoring the virus on target host cell membrane (Coutard et al., 2020). Based on homology to SARS-CoV, it has been reported that SARS-CoV-2 requires a host cell protease to achieve infectivity and spread (Hoffmann et al., 2020). After binding to the ACE2 receptor, the S2 protein is proteolytically activated by transmembrane serine protease 2 (TMPRSS2) in order to enter the host cell (Matsuyama et al., 2020).

COVID-19 presents with a wide spectrum of clinical severity, which ranges from a mild pneumonia to a severe disease that could result in acute respiratory distress syndrome disease-like (ARDS) (Guan et al., 2020; Wu and McGoogan, 2020). The ARDS-like feature in COVID-19 is notably different from that seen in septic patients (Yang et al., 2020). The main clinical laboratory findings associated with a poor outcome are lymphopenia, abnormal liver function test, raised serum levels of ferritin and C-reactive protein, and DDI (Shi et al., 2020; Wu et al., 2020; Zhou et al., 2020). High plasma DDI level is consistently advocated as a major predictor of mortality, which suggests that abnormal coagulation plays a key role the pathogenesis of COVID-19 (Chen et al., 2020; Grasselli et al., 2020; Tang et al., 2020a,b; Wang D. et al., 2020).

## Coagulation and Fibrinolysis: (See Figure 1)

The extrinsic pathway is triggered by tissue injury, which increases endothelial expression of activated tissue factor (aTF), which in turn activates FVII and the subsequent activation of FX, and formation of the aTF-FVIIa-FXa complex. This complex and the generated thrombin possess the ability to induce intracellular pro-inflammatory signaling via protease-activated receptor 1 and 2 (PAR 1 and 2) (Montagnana et al., 2017). On the other hand, the protein C complex, which comprises thrombin-thrombomodulin and activated protein C, deactivates FVIIIa and FVa (acceleration factors), and results in deceleration of the coagulation process (Vatsyayan et al., 2014; Yau et al., 2015). This anti-coagulation pathway (protein C complex) requires an intact vascular endothelium, which expresses the endothelial cell protein C receptor (EPCR) (Palta et al., 2014; Swieringa et al., 2018). A damaged endothelium frees EPCR (soluble EPCR), which avidly binds to the free activated protein C complex and loss of its anticoagulant moiety, promotes hypercoagulability (Ducros et al., 2012). In addition, the EPCR-Protein C complex exerts a cytoprotective effect under normal conditions (Zelaya et al., 2018). This complex activates PAR1 signaling to generate anti-inflammatory and anti-apoptotic effects (Mosnier et al., 2007; Mohan Rao et al., 2014).

**FIGURE 1:**
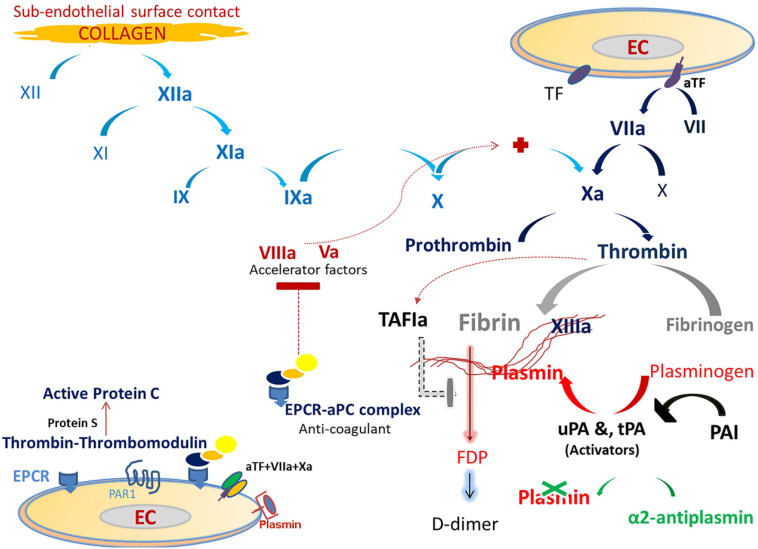
The figure displays the coagulation and fibrinolysis processes. Traditionally, the extrinsic pathway of the main coagulation cascade depends on the release of tissue factor (TF). Injured endothelial cells (EC) by any cause results in increased expression of TF in the cell membrane. This increased expression activates FVII, which then results in TF-FVIIa binding and activating FX. FX is also activated via the intrinsic pathway (collagen path). TF-FVII-FXa is the coagulation initiation complex which binds to EC and stimulates the generation of thrombin from prothrombin. Thrombin triggers many processes, which include fibrin generation, FXIII activation, and activation of protein C. FXIIIa polymerizes fibrin to form the final stable clot. Thrombin binds to the endothelial Protein C receptor (EPCR) and combines with thrombomodulin to activate protein C (aPC) and generate the EPCR-aPC complex. This complex has anti-coagulation activity by blocking the procoagulant factors, FVa and FVIIIa (accelerator factors) and a cytoprotective effect via the protease-activated receptor 1 (PAR1). Fibrinolysis (clot solubilization) is triggered in the presence of fibrin. The conversion of circulating zymogen plasminogen to plasmin is done by endothelial enzymes and the tissue and urokinase plasminogen activators (tPA and uPA, respectively). Plasmin, a key player in fibrinolysis, is a serine protease which cleaves fibrin to generate fibrin degradation products (FDP). Plasminogen activator-inhibitor (PAI) blocks both tPA and uPA. The main plasmin inhibitor is α2-antiplasmin, which is a potent free plasmin (not bounded to fibrin) scavenger. In addition, TAFI (thrombin-activatable fibrinolysis inhibitor) attenuates plasmin generation.

The activated fibrinolytic system on endothelial cells is crucial for dissolving the fibrin clot and facilitating tissue repair. Plasmin, a serine protease, is the key-player in this system and is responsible for degrading fibrin (Olson, 2015; Iba and Levy, 2018).

## COVID-19 and Coagulation–Fibrinolysis Dysfunction

Reports on patients with COVID-19 emphasize the presence of increased thrombosis and fibrinolysis and less bleeding diathesis (Huang et al., 2020; Tang et al., 2020b). An atypical disseminated intravascular coagulation (DIC) is also seen in COVID-19 (Klok et al., 2020) as thrombocytopenia, hypofibrinogemia, hemolytic anemia, and bleeding are under-represented (Fox et al., 2020; Han et al., 2020; Tang et al., 2020b). Of all the features, the most prominent prognostic factor in patients with COVID-19 is the high plasma DDI levels (Iba et al., 2017; Tang et al., 2020b; Zhou et al., 2020).

Recent clinical observations provide evidence that COVID-19 patients are at high VTE and mortality risks, and anticoagulant therapy might improve their prognosis (Paranjpe et al., 2020; Song et al., 2020; Tang et al., 2020a; Wu et al., 2020; Yin et al., 2020). General agreement exists on the need for thromboprophylaxis in majority of COVID-19 patients, and some suggest that this treatment should be continued after hospital discharge (Kollias et al., 2020).

Coagulation abnormalities include a prolonged prothrombin time, low antithrombin activity, and increased fibrinogen and DDI levels. However, the mechanisms of abnormal coagulation and fibrinolysis in COVID-19 patients are unknown (Han et al., 2020; Tang et al., 2020b).

The finding of a very high plasma DDI level is a hallmark for *hyper-fibrinolysis in COVID-19.* The strong association between high plasma DDI levels and the poor outcome raises several thoughts. Anticoagulation therapy barely reduces mortality, except in a small subgroup of patients, whose plasma DDI levels were six times greater than normal levels (Tang et al., 2020b). Alteplase is a fibrinolytic agent and when administered to patients with COVID-19-related ARDS, all died despite improvements in their oxygenation indices (Wang J. et al., 2020). This treatment was based on the results of animal studies which found that fibrinolytic drugs might improve lung function and alleviate inflammation in ARDS-like animal models (Hardaway et al., 1990; Liu et al., 2018). The present evidence does not support the use of fibrinolytic drugs in COVID-19 patients with ARDS. We can deduce that hyper-fibrinolysis plays a key role in the high pathogenicity and infectivity of SARS-CoV-2.

## Comprehensive Role of Fibrinolysis in COVID-19 Pathogenicity: (Figure 2)

SARS-CoV-2 binds with high avidity to the ACE2 receptor. This enzyme exerts a protective function on endothelial cells and pneumocytes, (Tikellis and Thomas, 2012) by virtue of its anti-inflammatory, anti-thrombin and anti-oxidant activity (Pai et al., 2017; Bavishi et al., 2020). A reduction in the protective effects of ACE2, as in aging, diabetes mellitus, and cardiovascular diseases, results in cellular damage and harmful consequences, with increasing oxidative stress and thrombosis (Tikellis and Thomas, 2012). Of note, administering recombinant ACE2 to ACE-deficient mice with induced lung injury protects them from developing an ARDS-like syndrome (Imai et al., 2005). The high mortality in old COVID-19 patients with comorbidities associated with endothelial dysfunction, indicates that this protective effect of ACE2 may be essential for survival (Patel and Verma, 2020; Sunden-Cullberg, 2020). Accordingly, it has been suggested that COVID-19 patients could be treated with human recombinant soluble ACE2 (Batlle et al., 2020; Kruse, 2020). Despite evidence for increased expression of ACE2 in patients with cardiovascular disease who are treated with ACE inhibitors (ACE-I) and angiotensin receptor blockers, the actual impact of these drugs on COVID-19 was reported to be controversial (Danser et al., 2020; Mancia et al., 2020). Of note, ACE-Is have an anti-fibrinolytic effect in humans, (Tiryaki et al., 2010) and recent guidance recommends continuing these drugs in patients with cardiovascular diseases.

**FIGURE 2:**
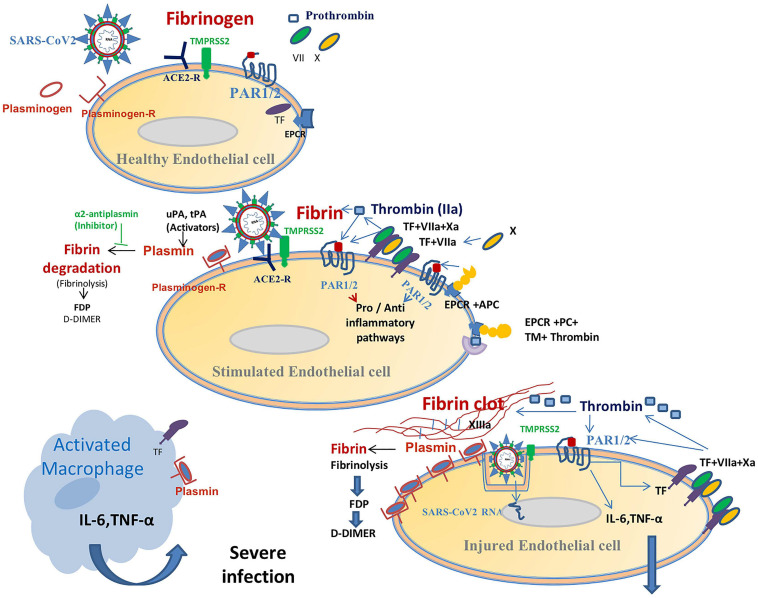
This figure displays a suggested relationship between coagulation and inflammation in patients with a SARS-CoV-2-infection. Spike proteins in the coronavirus’ envelope first bind to angiotensin-converting enzyme 2 (ACE2) receptors on the surface of epithelial cells (e.g., EC, endothelial cell). Type II transmembrane serine protease (TMPRSS2) then cleaves the spike protein in order to facilitate viral entry. The SARS-CoV-2 virus damages EC and promotes activation of the coagulation cascade (see Figure 1) via the TF-FVIIa (tissue factor pathway) and boosts fibrin generation (hyper-coagulation state). This hyper-activation of the fibrinolytic system results in increased plasmin production. Plasmin binds to the EC-plasmin receptor and degrades fibrin that leads to high plasma DDI levels. Membranous plasmin, which is a potent serine protease, could cleave the virus’ protein S hereby aiding its entry into host cells. This shared viral cleavage function with TMPRSS2 contributes to the increased infectivity of the virus and facilitates its spread (this function has been established in many viral infections, see text). In addition, over-production of plasmin (increased plasmin-receptor occupancy) activates the protease-activated receptor 1 and 2 (PAR 1, 2) signaling pathways which are involved in inflammation and immune responses. The EPCR-protein C complex is probably overwhelmed by hypercoagulability, thereby losing its cytoprotective activity. This virus-related pro-coagulation-inflammatory state also involves macrophages. This pathophysiologic paradigm explains the increased infectivity (and replication?) of the virus and the association between high D-dimer production and the cytokine storm (mainly IL-6, IL-10, and TNF) in COVID-19.

Transmembrane serine protease 2 has pivotal role in the infectivity of SARS-CoV-2. The cleavage of the coronavirus’ S protein by TMPRSS2 is not exclusive for SARS-CoV-1 and 2 (Hoffmann et al., 2020; Matsuyama et al., 2020). Other viruses enter host cells by utilizing this pathway, such as the influenza H1N1 and herpes viruses (Hamming et al., 2004; Matsuyama et al., 2020). Results from *in vitro* studies showed that TMPRSS2 inhibition does not completely block virus entry into host cells (Tang et al., 2020c; Zhang et al., 2020). Camostat mesylate, a potent serine protease inhibitor which efficiently inhibits TMPRSS2, is currently under clinical investigation for reducing the virus infectivity in COVID-19 patients (Kawase et al., 2012; Hoffmann et al., 2020).

Although TMPRSS2 is the major enzyme which facilitates the entry of SARS-CoV-2 into the host cell, other serine proteases possess this activity (Hoffmann et al., 2020; Walls et al., 2020). The serine proteases, trypsin, elastase and furin, can cleave S protein in the viral envelope of SARS-CoV and MERS-CoV (Ji et al., 2020; Rabi et al., 2020; Tang et al., 2020c). Furin is a part of the *trans-*Golgi network and is highly expressed on endothelial and pneumocyte cells, and it has been recently reported that it also cleaves SARS-CoV-2 (Braun and Sauter, 2019; Lukassen et al., 2020; Walls et al., 2020). Furin can also cleave the S protein of non-coronaviruses, such as the West Nile, Zika, and respiratory syncytial (RS) viruses (Millet and Whittaker, 2015; Coutard et al., 2020; Lukassen et al., 2020).

Plasmin, which is bound to plasmin-Receptor located on cell membranes (Figure 2), possesses furin-like cleavage activity (Miles et al., 1986; Kam et al., 2009; Zhao et al., 2020). Plasmin’s cleavage activity was first described for the influenza H1N1 virus (Goto et al., 2001; Murakami et al., 2001). However, plasmin’s cleavage activity (furin-like) on SARS-CoV-2 requires elucidation (Millet and Whittaker, 2015).

Therefore, cell entry of the virus depends on the specific binding to ACE2 and cleavage by TMPRSS2, but this latter step can be replaced by other serine proteases, such as plasmin (Kawase et al., 2012; Hoffmann et al., 2020; Zhang et al., 2020).

Findings from autopsies of severely affected COVID-19 patients include the presence of abundance of fibrin deposition, (Fox et al., 2020) which requires increased plasmin activity. In addition to its cleavage activity on viruses, plasmin can activate human macrophages promoting production of pro-inflammatory cytokines, such as IL-6, IL-8, IL-10, and TNF (Li et al., 2007). An increased plasma IL-6 level is a marker of the “cytokine release syndrome” in COVID-19 and is associated with poor outcome (Henry et al., 2020). To summarize thus far, high plasmin activity could participate in the perpetuity of virus infectivity and contribute to the excessive inflammatory and immune responses in COVID-19.

Acute respiratory distress syndrome is the most challenging clinical finding and the leading cause of death in patients with COVID-19-associated pneumonia (Wu et al., 2020). The pneumocytes and endothelial cells in the pulmonary alveoli share similar protective biologic mechanisms (Tikellis and Thomas, 2012; Nova et al., 2019). Some patients with COVID-19 present in a procoagulant state with a catastrophic microvascular injury in their lungs (Fox et al., 2020; Magro et al., 2020). The coronaviruses, MERS-CoV, SARS-CoV, and SARS-CoV-2, target cells with high expression of ACE2 and TMPRSS2, such as endothelial cells and pneumocytes (Glowacka et al., 2011). ACE2 has an important protective function in these cells. The lung injury in SARS is reported to be dependent on the balance between coagulation activity and the extent of fibrinolytic process (Gralinski et al., 2013). It is also known that plasminogen-plasmin activity is increased in ARDS (Spadaro et al., 2019). The levels of procoagulant components, in the bronchoalveolar lavage of patients with ARDS, such as plasmin and fibrinolytic degradation products, are markedly higher than in those without ARDS (Fuchs-Buder et al., 1996). A suggestion for the role of the fibrinolytic system in the genesis of ARDS in COVID-19 patients, (Idell, 2003; Spadaro et al., 2019) is supported by a the results of an investigation in plasminogen activator inhibitor-1 (PAI-1) deficient mice. The results indicate that the tPA and uPA contribute to the development the lung injury in coronaviruses infection, and PAI has a protective function in this condition (Gralinski et al., 2013). This suggest that “partial” inhibition of the hyper-fibrinolytic process in COVID-19 might mitigate the development of ARDS. An activated coagulation-plasmin-fibrin pathway in ARDS triggers a various protease secretion, such as elastase, and strong cytokine response, which is manifested by activated leukocytes and macrophages (Gralinski et al., 2013; Spadaro et al., 2019). The cytokine release syndrome has not yet been fully characterized in patients with COVID-19 (Pedersen and Ho, 2020). However, there is evidence that the high viral load in the lungs of COVID-19 patients is associated with an acute inflammatory response comprising epithelial cells and activated macrophages, which are largely responsible for the secretion of the cytokines, such as TNF, IL-6, IL-8, IL-1β, and CXCL10 (Freeman and Swartz, 2020).

### Virus-Related Coagulation-Inflammation Interaction

Viruses affect the hemostatic system via activation/deactivation of platelet aggregation, coagulation, and fibrinolysis (Goeijenbier et al., 2012; Koupenova et al., 2018). There is an increasing body of evidence which shows that a viral infection orchestrates a collaborative process which connects coagulation with the inflammatory response (Goeijenbier et al., 2012). Viral infection elicits an inflammatory reaction, which in turn activates the coagulation system (Opal, 2003). Frequently, viral-related hemostasis elicits a procoagulant-thrombotic effect, such as that seen in cytomegalovirus, hepatitis C and HIV infections (van Dam-Mieras et al., 1992; Chiappetta et al., 2016). In contrast, ebola, dengue, and other hemorrhagic viruses, which can also cause endothelial damage, are associated with increased anti-coagulant effects and fatal hemorrhage (Schnittler et al., 1993; Mahanty and Bray, 2004). Viruses which damage endothelial cells can promote the generation of the *TF-VIIa-Xa-EPCR* complex (procoagulant path, Figures 1, 2), which is able to activate PAR2 and trigger an innate immune response (Zelaya et al., 2018). During viral infection, PAR2 activation provokes the toll-like receptor 4 (TLR 4) to modulate the inflammatory response (Antoniak and Mackman, 2014). On the other hand, some viral infections can increase thrombin production and activate the EPCR-aPC complex, which in turn stimulates PAR1 signaling to exert a cytoprotective effect (Mosnier et al., 2007; Antoniak and Mackman, 2014).

In summary, the resultant coagulation abnormalities in viral infections depends on the effect of the virus on the balance between the pro- and anti-coagulant pathways. A virus which mainly activates the procoagulant and fibrinolytic systems could induce in a severe inflammatory response. A virus which activate the anti-coagulant pathway i.e., could induce a mild inflammatory response (Mosnier et al., 2007; Antoniak and Mackman, 2014; Nieman, 2016).

For example, patients with dengue hemorrhagic fever produce antibodies against the virus, which can activate plasminogen and fibrinolysis and contributes to bleeding diathesis (Chuang et al., 2016). In contrast, SARS-CoV-2 causes hyper-fibrinolysis without any significant bleeding (Chen et al., 2020; Han et al., 2020).

#### Overview of COVID-19 Treatment

Although several therapeutic agents have been evaluated for the treatment of COVID-19, none have yet been shown to be efficacious (Sanders et al., 2020). The similarity in clinical features of coronaviruses infections offers therapeutic modalities based on SARS and MERS epidemics to clinicians. However, results on the efficacy of reported treatments in SARS and MERS are controversial (Stockman et al., 2006; Morra et al., 2018).

Lopinavir-ritonavir, an aspartate protease inhibitor combined with a CYP450 inhibitor for increasing its half-life, is reported as having no beneficial effects in COVID-19 patients (Cao et al., 2020). Ribavirin, a nucleotide analog, which blocks the viral RNA-dependent RNA polymerase, is also reported as having no beneficial effect (Morra et al., 2018). Remdisivir, a potent RNA polymerase inhibitor and whose use was compassionate, is reported to be effective in shortening the time to recovery in COVID-19 patients (Gordon et al., 2020; Grein et al., 2020).

The antimalarial drugs, chloroquine and hydroxychloroquine, which inhibit lysosomal activity and autophagy, have beneficial immunomodulatory effects (Schrezenmeier and Dorner, 2020). Hydroxychloroquine blocks the endosomal entry of SARS-CoV-2 into host cells and reduces cytokine production *in vitro* (Sinha and Balayla, 2020). Moreover, it has been reported that hydroxychloroquine (a) is not effective in preventing the development of COVID-19 after a moderate to high-risk exposure in out-patients and (b) does not affect the course of the disease in hospitalized patients (Boulware et al., 2020; Cavalcanti et al., 2020; Gautret et al., 2020; Geleris et al., 2020; Vanden Eynde, 2020).

The presence of high plasma IL-6 levels in COVID-19 patients may justify the use of tocilizumab, a monoclonal IL-6 receptor antibody, and offer a protective effect against the cytokine storm. However, its effect on virus replication and infectivity is doubtful (Biran et al., 2020; Buonaguro et al., 2020; Price et al., 2020). More recently, Japanese authors suggested treating COVID-19 patients with heparin and nafamostat mesylate, a synthetic serine protease inhibitor, which possesses anti-trypsin and anti-fibrinolytic effects (Asakura and Ogawa, 2020). Nafamostat is also being investigated because of ability to block MERS-CoV entry into host cells (Yamamoto et al., 2016).

### Mechanism-Based Proposed Treatment: (see Figure 2)

In light of the present need, it enables the use of an unconventional treatment for COVID-19 patients. Since a procoagulant state and hyper-fibrinolysis co-exist in COVID-19 (Tang et al., 2020b), we assume *that the increased fibrinolysis boosts the infectivity of SARS-CoV-2* via the *plasmin-mediated pathway.* In addition, plasmin elicits a pro-inflammatory response by activating macrophages (releasing IL-6, and TNF) and increases PAR2-TLR4 signaling (Li et al., 2007; Antoniak and Mackman, 2014). Moreover, the increased mortality in COVID-19 is associated with conditions, which are associated with endothelial dysfunction, low ACE2 expression, and high circulating plasminogen levels (Tikellis and Thomas, 2012; Derhaschnig et al., 2013).

*We suggest that pharmacologic interventions whose aim is to reduce plasmin production may decrease virus infectivity and attenuate the associated inflammatory condition* in COVID-19 patients.

*Tranexamic acid* (TA) competitively inhibits the activation of plasminogen (via binding to the kringle domain), thereby reducing the conversion of plasminogen to plasmin, which in turn results in lowering circulating DDI levels. TA is used to treat individuals with bleeding diathesis and can be administered, intravenously, orally, or locally. It can also be administered by inhalation to control pulmonary hemorrhage. TA’s half-life is ∼2-3 h, and is mainly eliminated in urine (McCormack, 2012). The efficacy of the inhaled route of administration has been tested in patients with hemoptysis. The investigators reported that inhaled TA was effective and safe for hemoptysis resolution (Wand et al., 2018). Similar results were obtained in patients with hemoptysis who were treated with oral or intravenous TA (Solomonov et al., 2009; Prutsky et al., 2016).

Tranexamic acid is well tolerated and the occurrence of adverse effects, such as mild to moderate headache, muscle cramp and arthralgia, nausea, and diarrhea, are uncommon (Freeman et al., 2011). While inhibition of fibrinolysis could increase thrombotic risk, there is no reported evidence for thrombo-embolism with the use of TA.

For the past two decades, TA has been used in combination with prophylactic anticoagulation (low dose warfarin, LMWH, and DOACs) in elderly patients undergoing major orthopedic surgery with high risk for thrombosis and hemorrhage (Wang et al., 2018; Tang et al., 2019). This combined therapeutic modality offers anticoagulant, anti-fibrinolytic and anti-inflammatory effects, thereby reducing thrombosis, bleeding and indices of inflammation (Gillette et al., 2013; Karampinas et al., 2019). *Hence, our suggestion is to use TA to treat patients with moderate to severe COVID-19-associated pneumonia*. TA could be administered through a systemic route or by using a closed-nebulizer (Wand et al., 2018). All COVID-19 patients should receive intensification of anticoagulant dosing (Barnes et al., 2020; Thachil et al., 2020; Zhai et al., 2020).

The thrombotic burden in COVID-19 patients increases with disease severity. Thus, the suggested intervention with TA should exclude critically ill patients. Future studies should address the timing of the intervention in light of the emerging data on SARS-CoV-2 dynamics and COVID-19 features (Sun et al., 2020; Zheng et al., 2020).

Administering *alpha 2-antiplasmin* (alpha 2-AP) is an alternative treatment to alleviate the respiratory distress of COVID-19 patients. Alpha 2-AP is a potent plasmin scavenger and is usually used as alpha 2-AP replacement therapy for patients with a homozygous alpha 2-AP deficiency. These patients are hemophilia-like and tend to bleed mainly after surgery and alpha 2-AP replacement therapy is the only efficient treatment for these patients (Saes et al., 2018). Therefore, this treatment should be reserved for those critically ill COVID-19 patients with low plasma alpha 2-AP levels.

## Summary

This proposed mechanisms and treatment modality are founded on a comprehensive review of reported investigations on the interactions between the coagulation-fibrinolysis and inflammation pathways in coronaviruses diseases. The severity of COVID-19-associated pneumonia places the patient at risk for irreversible ARDS and death. A balanced assessment of the risk-benefit ratio in a deteriorating patient with COVID-19 sometimes requires the implementation of an out-of-the-box treatment in the absence of an alternative proven treatment.

## Author Contributions

GJ: idea design and writing. AA: protein C expert and consultant. BB: writing and consultant as world expert in homeostasis. All authors contributed to the article and approved the submitted version.

## Conflict of Interest

The authors declare that the research was conducted in the absence of any commercial or financial relationships that could be construed as a potential conflict of interest.
